# Extracts from Minutes of Atlanta Medical Society

**Published:** 1867-09

**Authors:** 


### ARTICLE IV.

### *Extract from Minutes of Atlanta Medical Society.*

ATLANTA, JAN. 22, 1867.

On the call for report of cases, none being presented, the regular subject for discussion, *Post Partem hemorrhage*, was taken up.

*Dr. Douglas* said there seemed to be two particular causes leading to this difficulty—the presence of the placenta and simply inertia of the uterus.

*Dr. Word* rose merely to say that, in the treatment of this hemorrhage, he had been in the habit of using lead and opium, manipulations, ergot, and cold applications, and had never seen a fatal termination; had read in some work of the styptic effect of ipecac, but had not used it.

*Dr. W. F. Westmoreland* mentioned a case of hemorrhage following delivery before full period of utero-gestation, in which it was necessary to keep the finger in contact with the os, as the only means which seemed to promote contraction of the womb with certainty.

*Dr. Orme* thought it necessary always to guard against this accident by proper manipulations over the surface of the abdomen, so as to insure the proper contraction of the womb.

*Dr. Johnson* had found nothing so certain in the arrest of uterine hemorrhage after delivery, as ice introduced into the womb. He had no confidence in lead, but had sometimes found benefit from ergot; had, in an obstinate hemorrage, introduced a piece of ice nearly as large as a goose-egg, with prompt relief.

*Dr. O'Keefe* had never been very much troubled by this accident. His custom is to remove the placenta immediately after the child is handed over to the nurse, and by this prompt relief to the womb, encourage the natural contraction of the organ, thereby preventing the liability to hemorrhage.

*Dr. W. F. Westmoreland* asked permission to mention a case not exactly germane to the subject before the society. A lady to whom he had been called, now five months advanced in pregnancy, has been suffering with *flooding* for the past month, when assuming the erect posture. Pain preceded for several hours the commencement of the hemorrhage. He found but little trouble in arresting the discharge, requiring only to place the patient in the horizontal position, and give an opiate. He found opium alone equal to the combination of lead and opium.

*Dr. O'Keefe* thought the case just mentioned may be conducted safely to maturity, by confining the patient to the recumbent position. He had met with a similar case in which this system was enforced, and with the reward of a well developed child, carried the full time of utero-gestation.

*Dr. Johnson* remarked, that he had found the use of quinine highly beneficial in arresting hemorrhage, such as described.

---

TUESDAY EVENING, JAN. 29, 1867.

On the call for report of cases,

*Dr. O'Keefe* said he had none of importance to report, but wished to call the attention of the Society to Uterine Pathology. We are all, he said, called upon to treat cases where pain in the back and loins, leucorrhœa, hemorrhage, &c., &c., exist as the result of prolapsus, induration, engorgement, ulceration, ante-flexion, ante-version, and retroversion. In the treatment of such cases, the result is not generally very satisfactory. Where there are abrasions on the os or neck, resort is had to cauterants; where engorgement exists, to leeches; and if the general health be feeble, to tonics. If there be displacement, no attempt is made to correct it by the ordinary mechanical contrivances in use for this purpose, such as the uterine sound and pessaries; for such are found to be of no advantage. He desired to hear the experience of members of the Society in the management of such cases.

*Dr. W. F. Westmoreland* thought the experience of most physicians would sustain Dr. O'Keefe's opinion. He thought mal-position exists in at least half the females; and that in married women, there is no fixed position for the uterus. Congestion being the most frequent cause of these troubles, leeches are relied on in the treatment of most of them; even in ulceration, when the caustic fails to relieve the lesion, they may be applied with great advantage. Pessaries he regards as useless, to say the least of them.

*Dr. Boring* differed with Dr. W. in regard to cauterization. He has found much benefit from the use of it once a week, together with chlorate potassa and decoction of oak bark as injections. In order to restore the secretions, he generally resorts to mercury in the outset. Whenever these derangements occur, he believes some mal-position present.

*Dr. W. F. W.* rose to say Dr. Boring had misunderstood him. He did not object to cauterants, but give preference to local blood-letting.

*Dr. Douglas* rose to say, he thought all mechanical means for the cure of such affections unphilosophical. That he relies on general treatment and the plan proposed by Drs.

O'Keefe and W. F. Westmoreland. Such cases, in his opinion, are rarely cured perfectly.

*Dr. O'Keefe* referred to the obstacle these uterine derangements offered to conception, and the duty of the females under these circumstances.

*Dr. W. F. Westmoreland* thought sterility rarely depended upon displacement of the womb, but on its peculiar condition, and the deranged secretions washing out the spermatozoa.

*Dr. Word*, in well defined ulcers, can promise a cure generally, if the woman be otherwise healthy; but if the strength of the patient has been lost, failure will follow our efforts to cure. He prefers creosote to caustic silver, in most cases.

